# Recombinant mycobacterium tuberculosis fusion protein for diagnosis of mycobacterium tuberculosis infection: a short-term economic evaluation

**DOI:** 10.3389/fpubh.2023.1105857

**Published:** 2023-05-03

**Authors:** Zheng Liu, Sha Diao, Linan Zeng, Dan Liu, Xuefeng Jiao, Zhe Chen, Xiao Cheng, Xiaofeng Ni, Siyi He, Bin Wu, Deying Kang, Chaomin Wan, Rongsheng Zhao, Huiqing Wang, Lingli Zhang

**Affiliations:** ^1^Department of Pharmacy, West China Second University Hospital, Sichuan University, Chengdu, China; ^2^Evidence-Based Pharmacy Center, West China Second University Hospital, Sichuan University, Chengdu, China; ^3^NMPA Key Laboratory for Technical Research on Drug Products In Vitro and In Vivo Correlation, Chengdu, China; ^4^Key Laboratory of Birth Defects and Related Diseases of Women and Children, Sichuan University, Ministry of Education, Chengdu, China; ^5^West China School of Medicine, Sichuan University, Chengdu, China; ^6^West China School of Pharmacy, Sichuan University, Chengdu, China; ^7^Department of Pharmacy, Renji Hospital Affiliated with the School of Medicine, Shanghai Jiaotong University, Shanghai, China; ^8^Chinese Evidence-based Medicine Center, West China Hospital, Sichuan University, Chengdu, China; ^9^Department of Pediatrics, West China Second University Hospital, Sichuan University, Chengdu, China; ^10^Department of Pharmacy, Peking University Third Hospital, Beijing, China; ^11^Medical Simulation Centre, West China Second University Hospital, Sichuan University, Chengdu, China

**Keywords:** recombinant mycobacterium tuberculosis fusion protein, tuberculin pure protein derivatives, mycobacterium tuberculosis infection, economic evaluation, decision tree model

## Abstract

**Objectives:**

Recombinant Mycobacterium tuberculosis fusion protein (EC) was anticipated to be used for the scale-up of clinical application for diagnosis of Mycobacterium tuberculosis infection in China, but it lacked a head-to-head economic evaluation based on the Chinese population. This study aimed to estimate the cost-utility and the cost-effectiveness of both EC and tuberculin pure protein derivative (TB-PPD) for diagnosis of Mycobacterium tuberculosis infection in the short term.

**Methods:**

From a Chinese societal perspective, both cost-utility analysis and cost-effectiveness analysis were performed to evaluate the economics of EC and TB-PPD for a one-year period based on clinical trials and decision tree model, with quality-adjusted life years (QALYs) as the utility-measured primary outcome and diagnostic performance (including the misdiagnosis rate, the omission diagnostic rate, the number of patients correctly classified, and the number of tuberculosis cases avoided) as the effective-measured secondary outcome. One-way and probabilistic sensitivity analyses were performed to validate the robustness of the base-case analysis, and a scenario analysis was conducted to evaluate the difference in the charging method between EC and TB-PPD.

**Results:**

The base-case analysis showed that, compared with TB-PPD, EC was the dominant strategy with an incremental cost-utility ratio (ICUR) of saving 192,043.60 CNY per QALY gained, and with an incremental cost-effectiveness ratio (ICER) of saving 7,263.53 CNY per misdiagnosis rate reduction. In addition, there was no statistical difference in terms of the omission diagnostic rate, the number of patients correctly classified, and the number of tuberculosis cases avoided, and EC was a similar cost-saving strategy with a lower test cost (98.00 CNY) than that of TB-PPD (136.78 CNY). The sensitivity analysis showed the robustness of cost-utility and cost-effectiveness analysis, and the scenario analysis indicated cost-utility in EC and cost-effectiveness in TB-PPD.

**Conclusion:**

This economic evaluation from a societal perspective showed that, compared to TB-PPD, EC was likely to be a cost-utility and cost-effective intervention in the short term in China.

## 1. Introduction

Tuberculosis (TB) is a chronic disease that is caused by a Mycobacterium tuberculosis (MTB) infection and has become a serious and urgent public health problem in the world. The vast majority of infected people are said to be in a state of latent tuberculosis infection (LTBI) without exhibiting any signs or symptoms, 5–10% of whom will develop active tuberculosis (ATB) throughout their lives if they are out of the preventive intervention (1). The Global Tuberculosis Report 2021 issued by the WHO estimated that approximately one-third of the total global population, with an estimated 2 billion people, were infected with MTB in 2020, including more than 350 million in China alone. In 2020, the total incidence of TB in China was approximately 842,000, accounting for 8.5% of all estimated incident cases worldwide, ranking as the second highest country to bear the TB disease burden in the world (2). Ending the epidemics of TB by 2030 is one of the Sustainable Development Goals (SDGs) of the United Nations, and it is essential to reduce the infected population and control the source of new cases to achieve the above goals (3).

Currently, there is no gold standard test for direct identification of LTBI, and the basic principle of LTBI detection is to observe the TB-specific immune response in the human body to determine whether the patient is affected by an MTB infection or not. The broadly available tests for treating an MTB infection include the tuberculin skin test (TST), interferon-gamma release assays (IGRAs), and *in vitro* antigen–antibody assays (4). TST is an easy operation that does not not require any special equipment or a laboratory setup, but meanwhile, this test is prone to false-positives and poor specificity due to multiple antigenic components, especially in those patients who had received Bacillus Calmette–Guérin (BCG) vaccination. IGRAs and *in vitro* antigen–antibody assays have better sensitivity and specificity than TST, but they are more expensive and require the collection of human peripheral blood to be tested with specific instruments in the laboratory, which is not conducive to large-scale promotion and application.

To improve the testing efficiency, it is noteworthy that a novel diagnostic skin test based on specific MTB antigens has been developed and marketed in recent years, including Diaskintest (Generium Pharmaceutical, Moscow, Russia), C-Tb (Statens Serum Institut, Copenhagen, Denmark), and the Recombinant Mycobacterium Tuberculosis Fusion Protein (EC, Zhifei Longcom Biologic Pharmacy Co., Ltd., Anhui, China) (5). It consists of a 6-kDa early secreted antigenic target (ESAT-6) and a 10-kDa culture filtrate protein (CFP-10) specifically secreted by MTB, with fewer impacts from BCG vaccination or non-tuberculous Mycobacteria infection (6). Meanwhile, the application and operation procedure of these emerging technologies are similar to those of the purified protein derivative of tuberculin (TB-PPD) adopted in traditional TST, which might provide potential alternatives for the diagnosis of MTB infection.

Given the changed option available to patients for undergoing diagnostic tests in the public health system in China, the available evidence for optimal and affordable strategies for treatment of Mycobacterium tuberculosis infection is essential to policymakers, medical personnel, and patients. However, a head-to-head economic evaluation was absent when comparing the novel EC and the traditional TB-PPD based on the Chinese population. Thus, this study aimed to estimate the cost-utility and the cost-effectiveness of EC and TB-PPD for the diagnosis of MTB infection in the short term from a societal perspective for providing evidence for a clinical decision.

## 2. Methods

### 2.1. Study framework

The framework of this study was set as follow:

(i) Target population: a high-risk population of MTB infection, including (a) a close contact with pathogenically positive TB patients; (b) immunocompromised people who were infected with human immunodeficiency virus (HIV), or those who received immunosuppressive therapy, or those who were recommended to receive detection and preventive treatment based on a Chinese expert consensus (7).
(ii) Perspective: a societal perspective.
(iii) Strategies and setting: EC [0.3 ml/vial (Anhui Zhifei Longcom Biologic Pharmacy Co., Anhui, China)] vs. TB-PPD [1 ml:50 IU (Beijing Xiangrui Biologicals Co. Ltd., Beijing, China)], applied in the hospital or in the community.
(iv) Time horizon: 1 year, without discount on costs and health outcomes.
(v) Outcome measures: the primary outcome was measured in quality-adjusted life years (QALYs) as utility, and the secondary outcome was measured in the diagnostic performance as effectiveness, including the misdiagnosis rate, the omission diagnostic rate, the number of patients correctly classified, and the number of tuberculosis cases avoided.

### 2.2. Cost-utility analysis

Based on literature review (8–10) and expert consultation, this study constructed a decision tree model using TreeAge Pro 2011 (TreeAge Software, Inc., Williamstown, MA, USA) to simulate the overall cost and health outcomes of the target population after the screening, diagnosis, and treatment over 1 year, and to evaluate the economics of EC and TB-PPD using cost-utility analysis.

#### 2.2.1. Model structure

**Figure 1:**
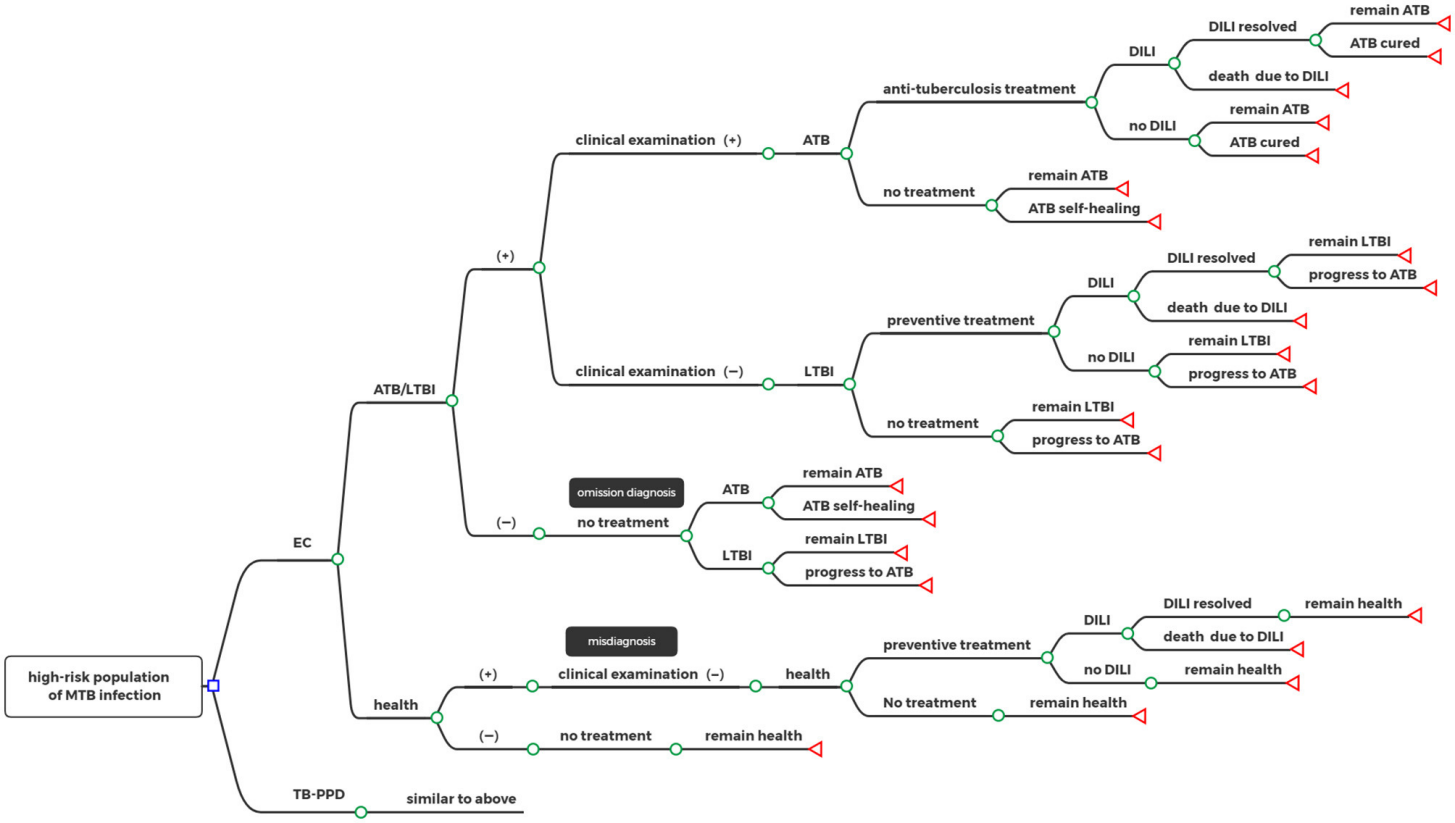
Decision tree model. □: decision nodes, °: chance nodes, ⊲: terminal nodes; EC, Recombinant Mycobacterium tuberculosis fusion protein; TB-PPD, tuberculin pure protein derivative; ATB, active tuberculosis; LTBI, latent tuberculosis infection; DILI, drug-induced liver injury.

The target population was subjected to an EC test or a TB-PPD test, respectively, where those who were screened negative with no need for any intervention or treatment, and those who were screened positive were diagnosed as having either ATB or LTBI and had to go through further clinical examination (including medical history, imaging, and etiology). Some omission errors in diagnoses and misdiagnoses happened in the test due to the absence of a gold standard for LTBI. According to individual willingness, patients diagnosed with ATB received anti-tuberculosis treatment or not, and those diagnosed with LTBI received preventive treatment or not. In addition, patients taking medication had a certain probability of drug-induced liver injury (DILI) during treatment. Based on different decisions, the status of patients had progressed, recovered, or remained the same. Details of the decision tree model can be seen in Figure 1.

#### 2.2.2. Model assumptions

The model assumptions were made as follows:

(i) All target populations were covered under BCG vaccination.
(ii) The medication compliance of the population receiving treatment was 100%.
(iii) The model parameters were adopted to suit the average level of the whole population since all age groups were susceptible to MTB.

#### 2.2.3. Model parameters

The parameters required for a model input included clinical and epidemiological probability, cost, and utility.

(i) Clinical and epidemiological probability: including sensitivity and specificity of EC and TB-PDD, the prevalence of ATB and LTBI, proportion of patients with ATB and LTBI receiving treatment, probability of DILI related to anti-tuberculosis treatment and preventive treatment, mortality of DILI, probability of progression from LTBI to TB with and without treatment, and the probability of cured and self-healing ATB.
(ii) Cost: including the test costs of EC and TB-PDD (i.e., the median price of the drug), cost of clinical examination, DILI treatment, ATB and LTBI treatments, consisting of direct medical costs (i.e., the registration fee, hospitalization fee, material fee, and drug fee), direct non-medical costs (i.e., transportation expenses, accommodation expenses, and food expenses), and indirect costs (i.e., the loss of salary for patients and their family caused by discontinuing school and sick leave).
(iii) Utility: including QALYs of patients with DILI, LTBI, ATB, cured, and self-healing ATB.

The cost estimates of EC and TB-PPD were taken from the China Pharmaceutical Information Database, and the remaining model parameters were sourced from published literature (11–33) (see Table 1). Preference was given to the most recent studies based on the Chinese population. All costs were updated to 2021 CNY using the Chinese Consumer Price Index. There was no time discounting of future costs and health outcomes as the period of the model was only 1 year. When more than one value of the same parameters was reported in multiple studies, the weighted mean was calculated as the baseline, and the maximum and minimum values, or baseline ± 5% if insufficient parameters, were included as the value range. For unavailable parameters, data were obtained through expert consultation or referred to relevant studies from other countries. All costs and probabilities are shown in Table 1.

**Table 1:** Model parameters.

**Parameters**	**Baseline**	**Range (Low–High)**	**Distribution**	**Reference**
**Clinical and epidemiological probability**			
Sensitivity of EC	0.9064	0.8750–0.9190	β	(6)
Specificity of EC	0.9272	0.8808–0.9736	β	(6)
Sensitivity of TB-PPD	0.9090	0.8860–0.9280	β	(6)
Specificity of TB-PPD	0.2658	0.2525–0.2791	β	(6)
Prevalence of ATB	0.0046	0.0043–0.0048	β	(11)
Prevalence of LTBI	0.1881	0.1373–0.2242	β	(12–14)
Proportion of ATB patients receiving treatment	0.9290	0.8190–0.9824	β	(11, 15, 16)
Proportion of LTBI patients receiving treatment	0.7130	0.6390–0.8631	β	(17–19)
Probability of DILI related to anti–tuberculosis treatment	0.0950	0.0380–0.1290	β	(20–22)
Probability of DILI related to preventive treatment*	0.0398	0.0100–0.0680	β	(23)
Mortality of DILI	0.0024	0.0024–0.0714	β	(24, 25)
Receiving treatment			
Probability of progression from LTBI to TB	0.0078	0.0003–0.0126	β	(26, 27)
Probability of cured ATB	0.9452	0.5710–0.9660	β	(28, 29)
No receiving treatment			
Probability of progression from LTBI to TB	0.0158	0.0058–0.0200	β	(27, 30)
Probability of self–healing ATB	0.0100	0.0100–0.2500	β	(19, 31)
**Cost**			
The test cost of EC	98.00	68.60–98.00	γ	(32)
The test cost of TB–PPD	136.78	67.80–158.00	γ	(32)
Clinical examination	178.93	125.28–232.57	γ	(8, 19)
DILI treatment	219.62	124.05–240.50	γ	(8, 19)
LTBI treatment	2158.05	1426.96–2889.14	γ	(19)
ATB treatment	21112.00	10556.00–63336.00	γ	(19)
**Utility**			
Health	1	/	/	/
Death	0	/	/	/
LTBI	0.9700	0.9500–1.0000	β	(19)
ATB	0.8200	0.6500–0.9300	β	(19)
ATB, cured or self–healing	0.9400	0.8700–1.0000	β	(19)
DILI^*^	0.6670	0.4000–0.8000	β	(33)

EC, Recombinant Mycobacterium tuberculosis fusion protein; TB-PPD, tuberculin pure protein derivative; ATB, active tuberculosis; LTBI, latent tuberculosis infection; DILI, drug-induced liver injury; ^*^data referred from outside China.

#### 2.2.4. Model outputs

The cost-utility ratio (CUR) and the incremental cost-utility ratio (ICUR) were calculated to compare the economics of the two strategies using the following formulas:


$$\begin{array}{l} \;CUR=\frac{\text{cost}}{\text{QALYs}}\\ ICUR=\frac{\text{incremental cost}}{\text{QALYs gained}} \end{array}$$

According to the recommendations of the China Guidelines for Pharmacoeconomic Evaluations (2020) and as per the definition of the WHO (34, 35), cost-utility was determined as assuming a willing-to-pay (WTP) threshold of 1–3 times per capita gross domestic product (GDP) of China in 2021 (80,976 CNY−242,928 CNY) (36) The increased cost was fully worthwhile and economical when ICUR was <80,976 CNY per QALY; the increased cost was acceptable when ICUR was more than 80,976 CNY per QALY but <242,928 CNY per QALY; while the increased cost was not economical when ICUR was more than 242,928 CNY per QALY (34).

### 2.3. Cost-effectiveness analysis

This study evaluated the diagnostic performance and short-term economics of EC or TB-PPD using cost-effectiveness analysis. Assuming that this study consisted of a hypothetical cohort of 10,000 participants, the misdiagnosis rate, the omission diagnostic rate, the number of patients correctly classified, and the number of tuberculosis cases avoided were calculated for each strategy as follows: (10, 37).

(i) Misdiagnosis rate = (1 - prevalence of ATB - prevalence of LTBI) × (1 - specificity) × 100%.
(ii) Omission diagnostic rate = (prevalence of ATB + prevalence of LTBI) × (1 - sensitivity) × 100%.
(iii) The number of patients correctly classified = the number of participants × (prevalence of ATB + prevalence of LTBI) × sensitivity.
(iv) The number of tuberculosis cases avoided = the number of participants × (prevalence of LTBI × probability of progression from LTBI to TB without treatment + prevalence of ATB) × sensitivity × basic reproduction number of TB.

The phase III clinical trial of EC, compared to that of TB-PPD, showed that no statistical significance was observed in the sensitivity between EC and TB-PPD (90.64 vs. 90.90%, mean difference [MD] = −0.26%, 95% confidence interval [Cl] = −2.39 to 1.36%, *p* > 0.05), but a statistical significance was presented in the specificity (92.72 vs. 26.58%, MD = 66.14%, 95% Cl = 60.76 to 71.52%, *p* < 0.05) (6). Thus, we directly compared the costs of EC and TB-PDD and regarded the lower one as the optimal strategy in terms of sensitivity and its derived indicators (the omission diagnostic rate, the number of patients correctly classified, and the number of tuberculosis cases avoided). Moreover, the cost-effectiveness ratio (CER) and the incremental cost-effectiveness ratio (ICER) were calculated to compare the economics of both EC and TB-PDD in terms of specificity and its derived indicator (misdiagnosis rate), using the following formulas:


$$\begin{array}{l} \;CER=\frac{\text{cost}}{\text{misdiagnosis rate}}\\ ICER=\frac{\text{incremental cost}}{\text{misdiagnosis rate reduced}} \end{array}$$

The data source was consistent with the cost-utility analysis. The result of cost-effectiveness analysis was only described in this study since there is no accepted WTP threshold for ICER.

### 2.4. Sensitivity analysis

One-way and probabilistic sensitivity analyses were conducted to assess the impact of uncertainty in these parameters and the robustness of the base-case analysis. In one-way sensitivity analysis, each parameter separately varied within the value range to explore the potential factors affecting the optimal strategy, and the results were shown in the tornado diagrams. In probability sensitivity analysis, Monte Carlo simulation was performed 1,000 times of multiple parameters simultaneously using the corresponding distribution to estimate the synthetic effect on the baseline results (38, 39), and the results were shown in the acceptability curves and the scatter plot.

### 2.5. Scenario analysis

As illustrated in the drug instruction, either EC or TB-PPD can be simultaneously administered to multiple subjects in the skin test, which was commonly applied in community screening. Thus, we performed the scenario analysis assuming that the medical institution charged the test at an average price of the available number of subjects, as per which EC can be administered for 3 people to the maximum and TB-PPD for 10 people to the maximum.

## 3. Results

### 3.1. Base-case analysis

As the base-case results are shown in Table 2, compared with the TB-PPD strategy, the EC strategy dominated with an ICUR of saving 192,043.60 CNY per QALY gained, and with an ICER of saving 7,263.53 CNY per misdiagnosis rate reduction. Additionally, in terms of the omission diagnostic rate, the number of patients correctly classified, and the number of tuberculosis cases avoided, the EC strategy offered more cost-saving than the TB-PPD strategy [the test cost of EC vs. the test cost of TB-PPD (98.00 CNY vs 136.78 CNY)].

**Table 2:** The results of the base-case analysis and the scenario analysis comparing EC to TB-PPD.

**Strategy**	**Cost (CNY)**	**QALYs**	**CUR (CNY/ QALYs)**	**ICUR (CNY/ QALYs)**	**Cost (CNY)**	**Misdiagnosis rate**	**CER (CNY/%)**	**ICER (CNY/%)**
**Base-case analysis**								
EC	579.0490	0.9915	584.0131	−192043.60 (Dominated)	980000	5.88%	166666.6667	7263.53 (Dominated)
TB-PPD	1539.2670	0.9865	1560.3315		1367800	59.27%	23077.4422	
**Scenario analysis**								
EC	513.7190	0.9915	518.1230	−180489.60 (Dominated)	326700	5.88%	55561.2245	−3556.85
TB-PPD	1416.1670	0.9865	1435.5469		136800	59.27%	2308.0817	

EC, Recombinant Mycobacterium tuberculosis fusion protein; TB-PPD, tuberculin pure protein derivative; QALYs, quality-adjusted life years; CUR, cost-utility ratio; ICUR, incremental cost-utility ratio; CER, cost-effectiveness ratio; ICER, incremental cost-effectiveness ratio.

### 3.2. Sensitivity analysis

#### 3.2.1. One-way sensitivity analysis

**Figure 2:**
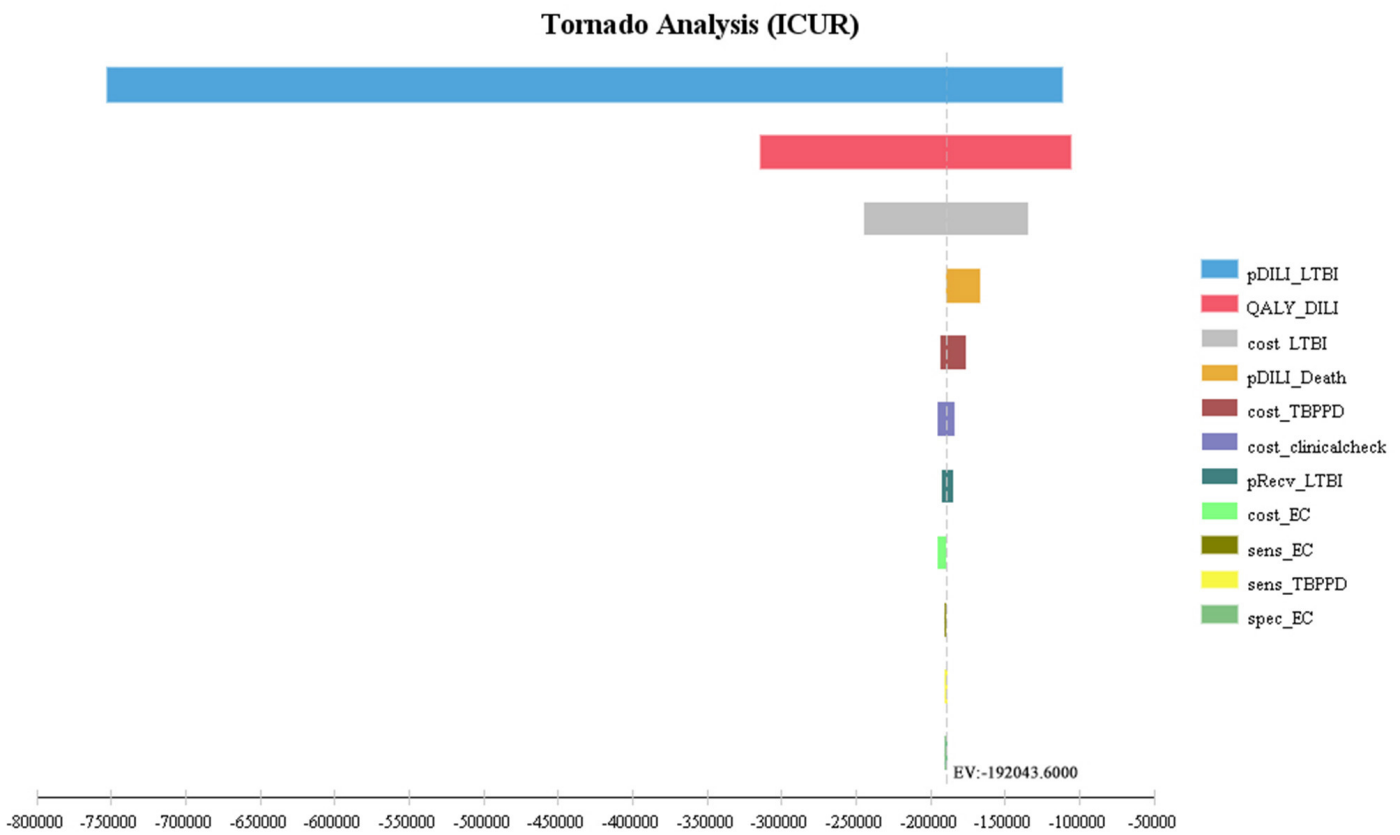
Tornado diagram of ICUR. pDILI_LTBI, probability of DILI related to preventive treatment; QALY_DILI, the QALYs of patients with DILI; cost_LTBI, the cost of LTBI treatment; pDILI_Death, probability of mortality of DILI; cost_TBPPD, the test cost of TB-PPD; cost_clinicalcheck, the cost of clinical examination; pRecv_LTBI, proportion of LTBI patients receiving treatment; cost_EC, the test cost of EC; sens_EC, sensitivity of EC; sens_TBPPD, sensitivity of TB-PPD; spec_EC, specificity of EC; EC, Recombinant Mycobacterium tuberculosis fusion protein; TB-PPD, tuberculin pure protein derivative; LTBI, latent tuberculosis infection; DILI, drug-induced liver injury.

**Figure 3:**
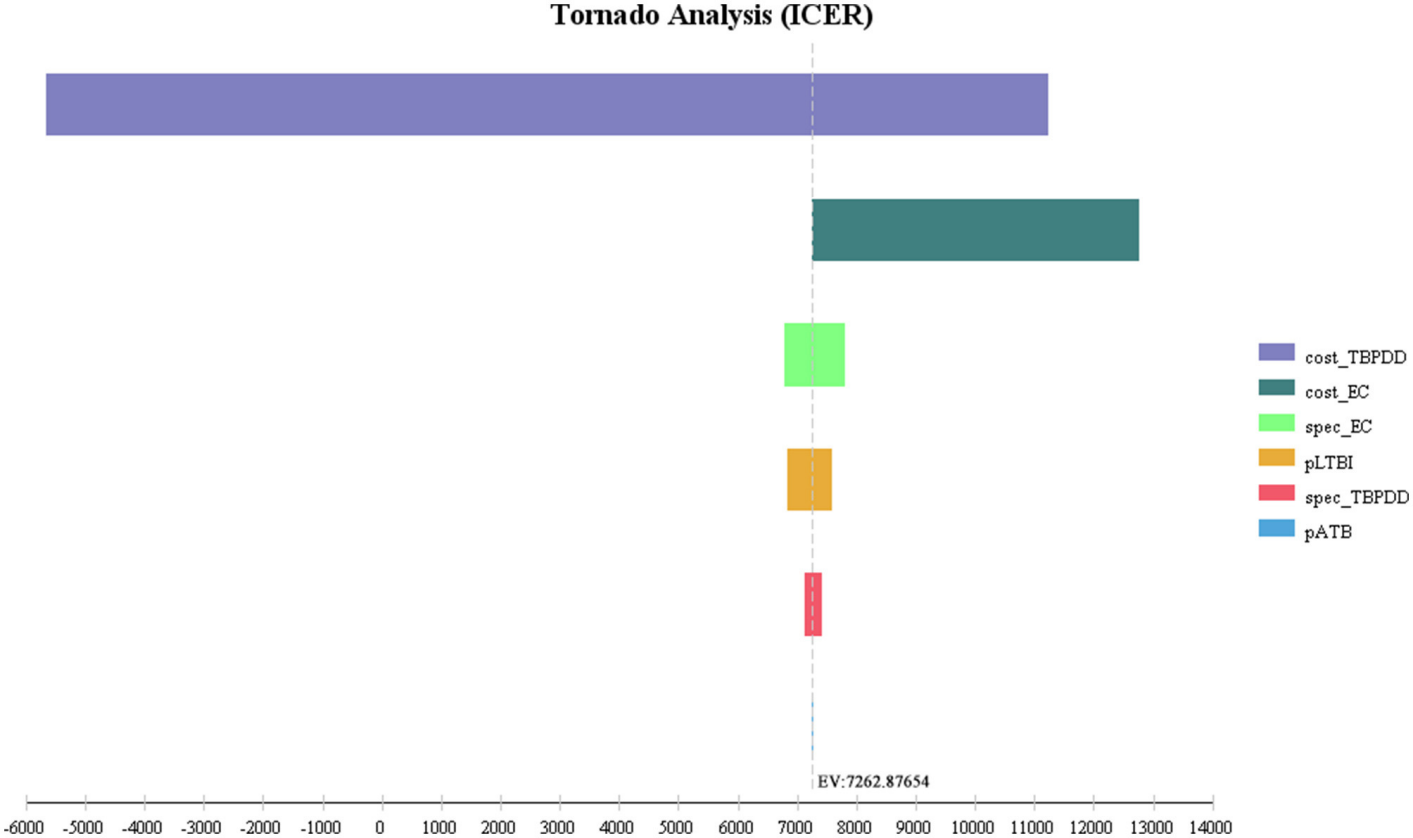
Tornado diagram of ICER. Cost_TBPPD, the test cost of TB-PPD; cost_EC, the test cost of EC; spec_EC, specificity of EC; pLTBI, prevalence of LTBI; spec_TBPPD, specificity of TB-PDD; pATB, prevalence of ATB; EC, Recombinant Mycobacterium tuberculosis fusion protein; TB-PPD, tuberculin pure protein derivative; ATB, active tuberculosis; LTBI, latent tuberculosis infection.

The first three factors with the greatest impact on the base-case analysis of ICUR were, in order, the probability of DILI related to preventive treatment, the QALYs of patients with DILI, and the cost of LTBI treatment. The ICUR of the above parameters varied within the value range were all <0, which was far less than the WTP threshold, suggesting that the EC strategy was more cost-utility than the TB-PPD strategy (Figure 2). Furthermore, those of ICER were, in order, the test cost of TB-PPD, the test cost of EC, and the specificity of EC. When the test cost of TB-PPD varied from 67.80 CNY to 158.00 CNY, the ICER would range from −5,655.98 to 11,237.04; when the test cost of EC varied from 68.60 CNY to 98.00 CNY, the ICER would range from 7,262.88 to 12,769.03; and when the specificity of EC varied from 0.8808 to 0.9736, the ICER would range from 7,810.84 to 6,786.76 (Figure 3).

#### 3.2.2. Probabilistic sensitivity analysis

**Figure 4:**
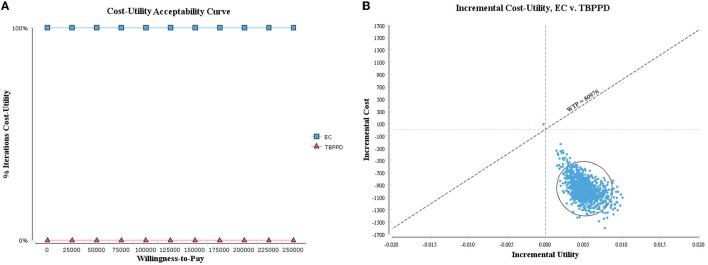
Probabilistic sensitivity analysis of ICUR. **(A)** Cost-utility acceptability curve; **(B)** cost-utility scatter plot.

**Figure 5:**
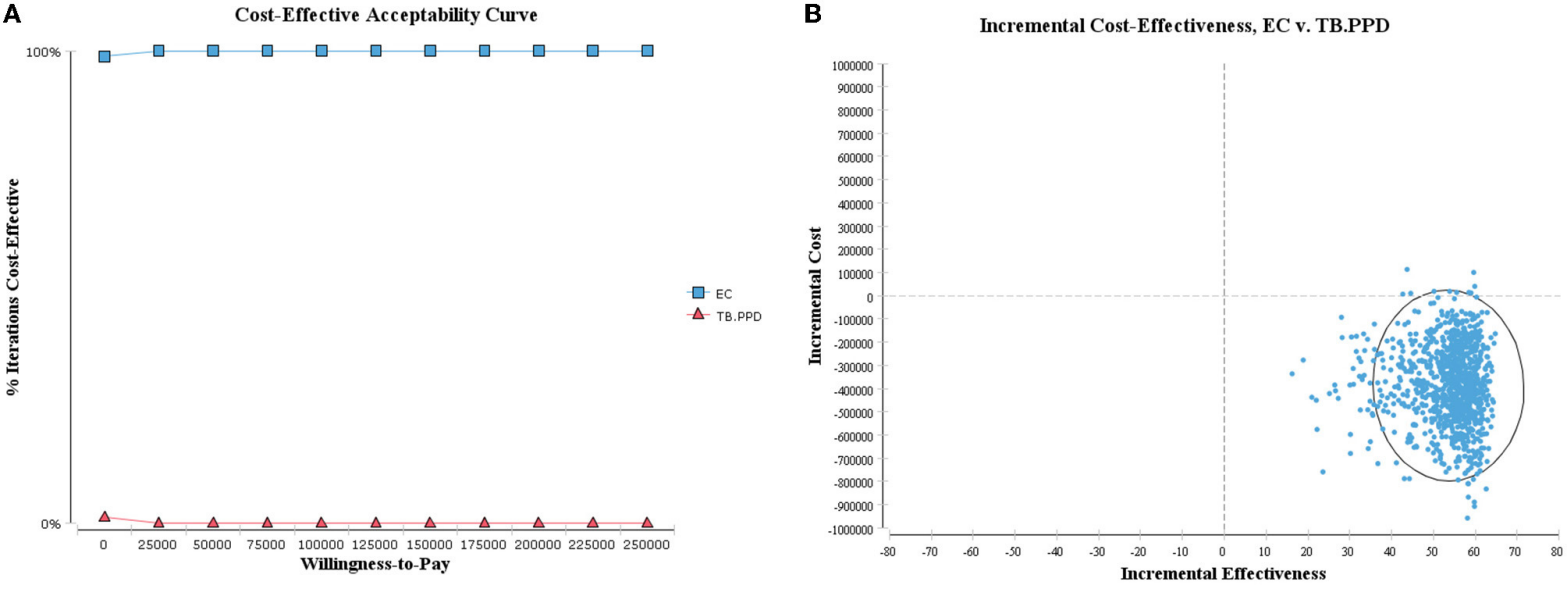
Probabilistic sensitivity analysis of ICER. **(A)** Cost-effectiveness acceptability curve; **(B)** cost-effectiveness scatter plot.

As shown in the cost-utility acceptability curve and scatter plot, the acceptable probability of EC was higher than that of TB-PPD within the WTP threshold (i.e., 242,928 CNY, three times GDP per capita), with 99.90% when WTP = 80,976 CNY/QALY (Figure 4). While in the cost-effectiveness acceptability curve and scatter plot, the vast majority of scatters were located in the fourth quadrant (99.00%), indicating that EC was a dominant strategy (Figure 5). The above results showed the robustness of the base-case analysis.

### 3.3. Scenario analysis

As the result of scenario analysis shown in Table 2, EC was also cost-utility when the medical institution charged at the average price of the available number of people, with an ICUR of saving 180,489.60 CNY per QALY gained, while EC rose to 3,556.85 CNY per QALY costing for each percent reduction in terms of the misdiagnosis rate in the assumed scenario. As regards the omission diagnostic rate, the number of patients correctly classified, and the number of tuberculosis cases avoided, TB-PPD presented more cost-savings than EC for a lower charge (13.68 CNY vs. 32.67 CNY).

## 4. Discussion

China is still one of the high-burden countries of TB, and early detection, early diagnosis, early reporting, early isolation, and early treatment of infected patients to reduce and avoid the epidemics of TB in the population are the main measures for current TB control in China (4). With the advancement in measures undertaken for TB prevention and control, policies and technologies have developed greatly. The approval and marketing of EC in China have provided a new technique for detection of MTB infection. The said technique can effectively identify MTB infection from BCG vaccination or other non-tuberculous mycobacterial infections compared with traditional TST (7). Due to the expansion of diagnostic techniques, we evaluated the short-term economics of EC and TB-PPD for the diagnosis of MTB infection based on the decision tree model from a societal perspective, and the results of this study showed that EC had more cost-utility and cost-effective advantages compared to TB-PPD.

As shown in the phase III clinical trial of EC, the sensitivity was not statistically significant but the specificity was statistically significant between EC and TB-PPD. Due to the absence of a gold standard for LTBI, there was still some inevitable error only relying on the test result to determine whether the human body was infected with MTB or not, and which probably resulted in a certain false diagnosis. The sensitivity and the specificity were associated with the false-negative and the false-positive, respectively. While among the target population in our study, LTBI patients with false-negative who missed further examination and treatment were much more likely to progress to ATB than those receiving treatment, thereby increasing the disease burden on society and patients. In addition, parts of healthy people with false-positive were misdiagnosed with LTBI, for those who selected to receive preventive treatment, had to afford additional treatment costs and increased risk of DILI that reduced their quality of life. Meanwhile, the current median price of EC is lower than that of TB-PPD (98.00 CNY vs. 136.78 CNY) in the market in China. Therefore, based on the above integrative factors, EC had a lower cost with a higher utility than TB-PPD due to its lower false-positive rate in the decision tree model.

According to the one-way sensitivity analysis, the first three factors affecting the baseline results of ICUR were the probability of DILI related to preventive treatment, the QALYs of patients with DILI, and the cost of LTBI treatment, among which the former two parameters were referred from outside China along with wide enough range for sensitivity analysis, but none of them reversed the base-case result. It indirectly indicated that the incidence of DILI had a certain impact on the economic burden for patients, which highlighted the need to pay attention to the occurrence of adverse events during treatment. The probabilistic sensitivity analysis, on the other hand, explored the synthetic effect of all parameters on the result under different WTP thresholds, and the scatters of ICUR values were overwhelmingly located in the fourth quadrant (i.e., lower cost and higher utility), suggesting the economic advantage of EC and further validating the robustness of the base-case analysis. In addition, for the sensitivity analysis of ICER, the magnitude of change in the test cost of TB-PPD was the most prominent, but it was not yet clear how it affected the result as there is no recognized cost-effectiveness threshold, and it is ultimately up to the payer to judge whether to accept the incremental cost or not. Therefore, the results of the cost-effectiveness analysis in this study were conservative and cautious.

Based on the field investigation conducted in the hospital and expert consultation, we learned that the hospitals commonly charged the skin test at the price of single specification of the drug, rather than at an average price of per person, which EC and TB-PPD can be used for performing tests in multiple subjects simultaneously illustrated in the drug instruction and label information. This is because there is no guarantee in the corresponding number of subjects present when each package is opened, otherwise it results in waste generation. Thus, we used the median price as the test cost parameter in the base-case analysis and meanwhile performed the scenario analysis to evaluate the influence of different charging methods on the results. Although EC charged a higher average price than TB-PPD for individual subjects in the assumed scenario, we found that EC still offered cost-utility with a lower total cost and better QALYs gained. However, it reversed and showed that EC had better effectiveness but spent more than TB-PPD in the misdiagnosis rate, for which we did not compare the economics due to lack of an accepted WTP threshold for ICER. In addition, the omission diagnostic rate, the number of patients correctly classified, and the number of tuberculosis cases avoided were similarly reversed for a lower charging of TB-PPD in the assumed scenario, indicating the impact of the cost of TB-PPD on short-term effectiveness.

To some extent, we made some innovations and improvements in this study. We evaluated the economics of the two current skin tests for diagnosis of MTB infection based on epidemiological data of the Chinese population for the first time. Then, we simulated the outcomes of the disease under different decisions within 1 year by constructing a decision tree model, to avoid problems of a long period and a high cost of clinical data collection, and difficulties in follow-up. Meanwhile, our study also shows several limitations. First, this economic analysis is still based on several assumptions. Considering the high proportion of BCG vaccinations in China, we assumed that the target population was vaccinated with BCG and extracted the data on specificity of this group from the phase III clinical trial rather than the placebo group, which may cover the actual cost-utility and cost-effectiveness of intervention strategies (40). Additionally, the model parameters were taken from the average of the all-age population, and the values of the same parameters for different populations were included as the range in the sensitivity analysis. Although the above parameters did not have any obvious impact on the base-case analysis, the results of this study may not be applicable to the special group, such as the elderly, children, and people who have not received BCG vaccination. For individual parameters referred from outside China, the results should be interpreted with caution. Second, the sensitivity and specificity in this study were directly derived from the phase III clinical trials of EC, rather than from a meta-analysis of current clinical research because of the insufficient literature about this novel test. Furthermore, we only performed the head-to-head comparison of the single test, and the combination of the different tests had not yet been included in this economic analysis, for which it was difficult to obtain the estimated parameters. The absence of the gold standard is still an unsettled issue in the current detection of MTB infection, rendering sensitivity and specificity to become critical factors in the test. It brought with it potential false diagnosis and unnecessary burden for patients, which required to be solved so as to improve the sensitivity and specificity of the diagnostic test to the greatest extent through technological innovation in the future.

## 5. Conclusion

In conclusion, our results indicate that, compared with TB-PPD, EC for diagnosis of MTB infection is a more cost-utility and cost-effective strategy in China, which may become a better choice in the current detection of MTB infection. This study also provides an important economic evidence for the diagnosis of MTB infection in the short term from a Chinese societal perspective for clinical decisions and policymakers.

## Data availability statement

The original contributions presented in the study are included in the article/Supplementary material, further inquiries can be directed to the corresponding authors.

## Author contributions

ZL implemented the project, interpreted the data, and drafted the original manuscript. SD, DL, and XC abstracted and analyzed the data. LNZ revised the manuscript. XFJ, ZC, XFN, and SYH searched and screened the literature. BW, DYK, CMW, and RSZ revised the study design and reviewed the final manuscript. LLZ and HQW conceived and designed the study and reviewed and approved the final manuscript. All authors have accepted responsibility for the entire content of this manuscript and approved its submission.
